# Intranasal delivery of dodecyl creatine ester alleviates motor deficits and increases dopamine levels in a 6-OHDA rat model of parkinsonism

**DOI:** 10.3389/fnagi.2025.1597263

**Published:** 2025-07-11

**Authors:** Clémence Disdier, Clara Lhotellier, Stéphanie Wagner, Emile Andriambeloson, Frédéric Théodoro, Alain Pruvost, Thomas Joudinaud, Henri Bénech, Aloïse Mabondzo

**Affiliations:** ^1^CERES BRAIN Therapeutics, ICM, Hôpital Pitié-Salpêtrière, Paris, France; ^2^Neurofit SAS, Illkirch, France; ^3^Département Médicaments et Technologies pour la Santé (DMTS), Université Paris-Saclay, CEA, INRAE, Gif-sur-Yvette, France

**Keywords:** Parkinson’s disease, 6-hydroxydopamine, dodecyl creatine ester, intranasal drug delivery, mitochondrial dysfunction, motor behavior

## Abstract

**Introduction:**

Creatine has been recognized not only as an energy buffer but also for its antioxidant, antiapoptotic, and anti-excitotoxic properties, making it of interest as a neuroprotective agent. Oral creatine monohydrate supplementation is ineffective due to poor brain and neuronal distribution and optimized forms of creatine deserve to be studied. Thus, dodecyl creatine ester (DCE), named CBT101, is a prodrug of creatine created for this purpose. When administered nasally it can follow the nose-to-brain pathway to deliver creatine to neuronal cells after passive diffusion across membranes. In this study, the therapeutic efficacy of intranasal DCE treatment was demonstrated in a 6-OHDA-intoxicated rat model, which is relevant to neurodegenerative diseases such as Parkinson’s disease.

**Methods:**

6-OHDA-intoxicated rats received DCE (13.3 mg/kg/day) or a vehicle intranasally for 5 weeks and were compared to a sham group. Imbalance in dopamine between the two hemispheres was assessed using the amphetamine-induced turning test after 3 weeks and sensorimotor performance using the beam walking test after 4 weeks, with ongoing treatment.

**Results and discussion:**

Five weeks after 6-OHDA intoxication, daily intranasal DCE treatment improved sensorimotor performance, striatal dopamine concentration, and modulated striatal pro-BDNF/BDNF balance and neurofilament expression both in plasma and in the striatum. These observations highlight DCE’s potential as a therapeutic strategy for neurodegenerative diseases characterized by energy deficiency and major mitochondrial dysfunction.

## Graphical abstract

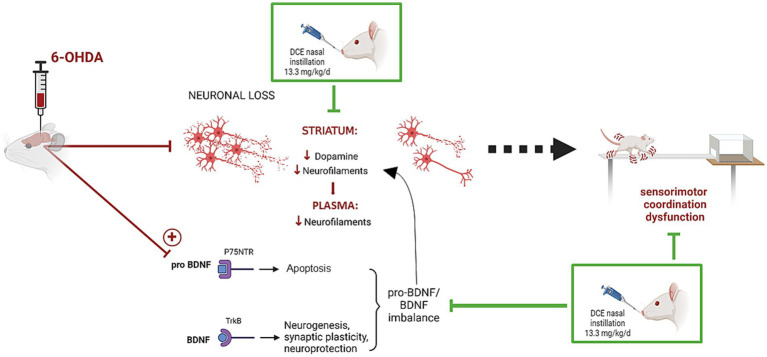

## 1 Introduction

Neurodegenerative disorders are debilitating brain diseases that lead to progressive impairments in behavior, movement, and cognition. At the cellular level, motor symptoms in neurodegenerative disorders are due to the death of neurons. However, neurodegeneration extends beyond neurons, with significant evidence also pointing to bio-energetic dysfunction and mitochondrial impairment as contributors to the disease’s pathogenesis (Klemmensen et al., 2024; Hirsch and Hunot, 2009; Macdonald et al., 2018; Lee et al., 2009).

Effective therapies and new drugs to prevent neuronal loss are still limited, highlighting the urgent need to develop innovative therapeutic approaches. Addressing this cellular energy deficit and improving mitochondrial function may provide therapeutic benefits for patients suffering from neurodegenerative diseases with similar underlying mechanisms.

Creatine (Cr), a natural amino acid crucial for mitochondrial energy metabolism and function, is produced not only by the liver, kidneys, and brain, but is also obtained through the diet (Forbes et al., 2022; Kreider and Stout, 2021; Roschel et al., 2021). It acts as an energy-buffering molecule, transported to the high-energy-demand sites such as brain and muscle cells, where it is phosphorylated into phosphoCr (PCr) thanks to the creatine phosphate shuttle. The latter moves inorganic phosphate from mitochondria into the cytosol to form PCr and supports cellular bioenergetics. During periods of high-energy consumption, the enzyme Cr kinase (CK) rapidly uses the phosphoryl group from phosphoCr to generate ATP from ADP. The PCr and Cr kinase pathway is highly effective for energy delivery within cells due to PCr’s superior diffusion capacity compared to ATP and the strategic localization of CK in energy production and consumption sites (Wallimann et al., 2011). Cr also possesses antiapoptotic, anti-excitotoxic, and direct antioxidative properties, both *in vitro* and *in vivo* (Genius et al., 2012; Lawler et al., 2002; Ogorman et al., 1997), which further support its potential interest as drug candidate. Thus, given the brain’s reliance on ATP and its tight coupling to the CK system, Cr supplementation is hypothesized to be a beneficial treatment for neurodegenerative diseases (Smith et al., 2014; Chang and Leem, 2023). However, a phase 3 clinical trial conducted by the administration of oral creatine monohydrate in symptomatic Parkinson’s disease (PD) or amyotrophic lateral sclerosis (ALS) patients was unsuccessful (Mo et al., 2017; Bender et al., 2008; Bender and Klopstock, 2016; Kieburtz et al., 2015; Groeneveld et al., 2003; Shefner et al., 2004).

Cr is transported into cells by the SLC6A8 transporter (Curt et al., 2015). Although SLC6A8 is present in microcapillary endothelial cells at the blood–brain barrier, it is absent in the surrounding astrocyte endfeet (Braissant and Henry, 2008). This explains the limited BBB permeability for peripheral Cr and partial reliance on endogenous synthesis within the brain (Beard and Braissant, 2010; Braissant, 2012). Consequently, the distribution of orally supplemented Cr monohydrate to brain cells, including astrocytes and more particularly to neurons, is restricted. In addition, the distribution to striatal neurons might be even more limited due to the almost absent expression of the Cr transporter at the membrane of those cells (Lowe et al., 2015). This is why it is hypothesized that recognized properties of Cr were not revealed in several neurodegenerative diseases such as PD or ALS during clinical trials (Mo et al., 2017; Bender et al., 2008; Bender and Klopstock, 2016; Kieburtz et al., 2015; Groeneveld et al., 2003; Shefner et al., 2004) due to poor brain and more particularly neuronal distribution of orally supplemented Cr (Bender and Klopstock, 2016).

Dodecyl creatine ester (DCE), a Cr prodrug developed for Cr transporter deficiency (Mabondzo et al., 2023; Trotier-Faurion et al., 2013; Trotier-Faurion et al., 2015; Ullio-Gamboa et al., 2019). When administered nasally, DCE reaches the deep brain structures via the nose-to-brain pathway, crosses cell membranes passively, and delivers Cr to neurons, even striatal neurons, in non-human primates (Disdier et al., 2025). Enhancement of Cr cerebral distribution with this dual strategy may also be of benefit in PD and other diseases involving energy deficits and mitochondrial dysfunction.

In this context, this study aims to demonstrate the therapeutic efficacy of DCE in the 6-hydroxydopamine (6-OHDA) neurotoxin rat model. This model is widely used in PD research (Lal et al., 2024). Sensorimotor assessment after 3 to 5 weeks of intranasal (IN) DCE treatment supports the rationale that bringing Cr into neurons alleviates PD-related symptoms. Three-fold labeling of the DCE molecule with stable isotopes (eg, DCE-δ3) was used to track the Cr originating from the DCE with respect to endogenous Cr. Investigation into cerebral effects revealed that the DCE treatment improved dopamine concentrations in the striatum. Modulation of BDNF and neurofilament expression in the striatum and plasma was also investigated as an underlying mechanism.

## 2 Materials and methods

### 2.1 Material

DCE-δ3 was synthesized from Cr-δ3, labeled threefold with stable hydrogen isotopes (3x^2^H), as described previously (Trotier-Faurion et al., 2013). Consequently, the labeling is only on the creatine side of the DCE molecule (Supplementary Figure 1).

Cr-δ3, Cr, Crn were from Sigma-Aldrich (Saint-Quentin Fallavier, France). Kollisolv MCT70 was from BASF (Ludwigshafen, Germany) and Montanox 80 from SEPPIC (Courbevoie, France).

All reagents for LC–MS/MS were of analytical grade. Waters acetonitrile, methanol, and formic acid were from VWR (Radnor, US). Ammonium acetate was from Sigma (Burlington, US), isopropanol from Honeywell, and acetic acid from Fluka.

### 2.2 DCE-δ3 formulation

The emulsion comprised 22.3% w/w of polysorbate 80 (Montanox 80), 19% w/w caprylic/capric triglyceride (Kollisolv MCT70) and 54.7% w/w of regular saline. The specified amount of DCE-δ3 (for a final concentration of 4% w/w) was first dissolved in the mix of oil and surfactant with magnetic stirring at room temperature (Stuart CB162, Bibbly Scientific, Nemours, France). The premix and the saline were preheated at 40°C. Finally, a fixed amount of preheated regular saline was added to the above mixture and stirred continuously for 10 min until production of a homogenous emulsion. The emulsion was stirred during the cooling phase and then stored at 4°C until use. The emulsion was prepared every 15 days and kept at 4°C.

### 2.3 Animal experiments

#### 2.3.1 Animals

**Figure 1:**
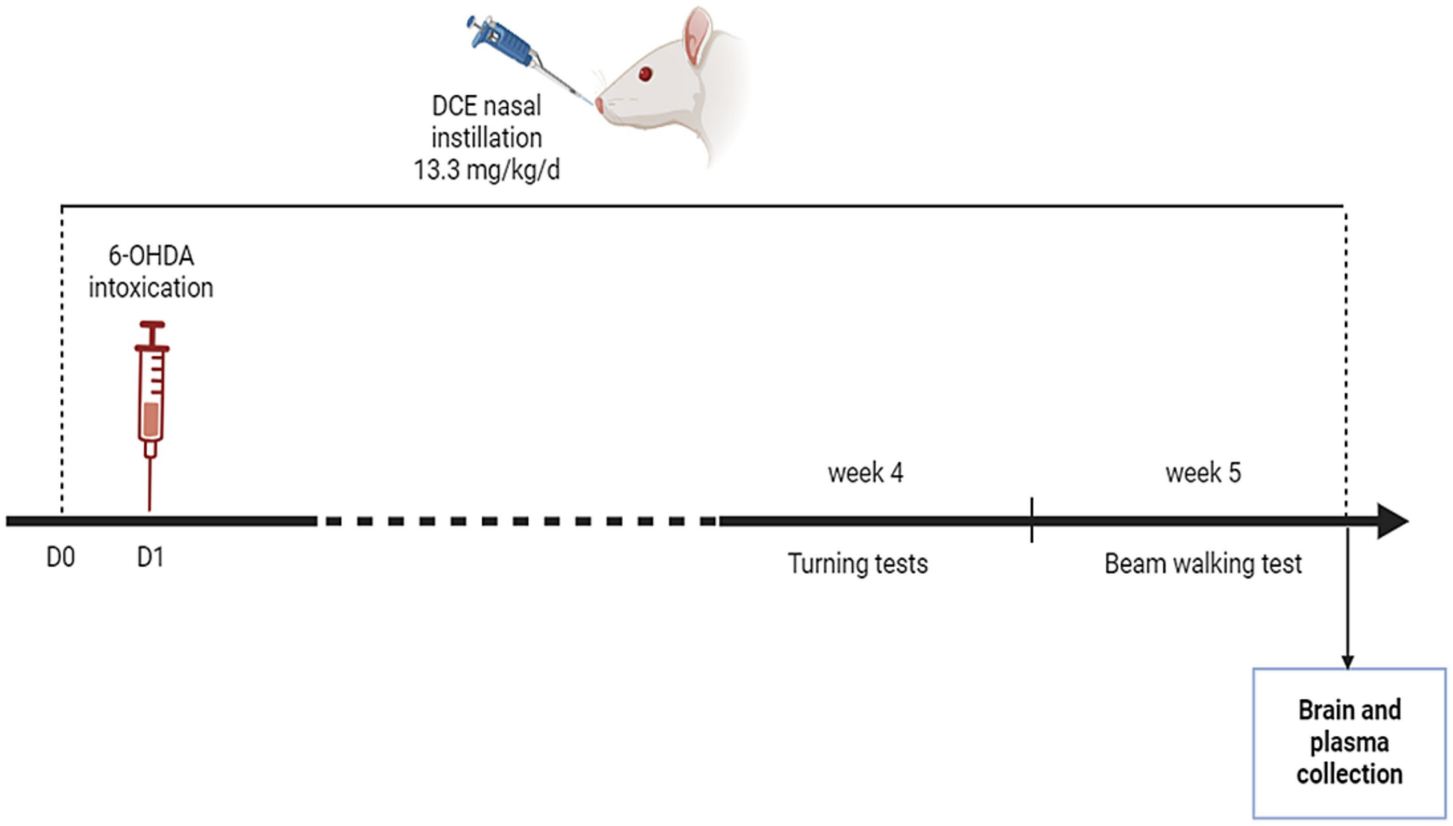
Experimental design. The Parkinson’s disease (PD) mouse model was induced by stereotactic 6-OHDA administration into the right medial forebrain bundle. The intranasal DCE-δ3 treatment was delivered to the rat starting on 1 day before 6-OHDA intoxication and daily for 5 weeks at a dose of 13.3 mg/kg/day. The sensorimotor tests were carried out in the 4th and 5th weeks. After that, the rats were sacrificed and biochemical and molecular analyses were carried out.

Male 8-week-old Sprague–Dawley rats weighing 250–300 g from Janvier (Le Genest St Isle, France) were housed in a controlled environment (temperature range, 22–24°C; relative humidity, 40–60%) under a reversed 12-h light and dark cycle with ad libitum access to food and water. The experimental procedure was approved by the external Ethics Committee (CEE35) registered at the French Ministry of Research. These animal experiments were performed at Neurofit SAS, Illkirch, France. The experimental design is schematized in Figure 1.

#### 2.3.2 6-OHDA lesion induction

Rats received an intraperitoneal injection of 15 mg/kg desipramine and a subcutaneous injection of 0.02 mg/kg buprenorphine 30 min prior to surgery. They were then anesthetized using a mixture of 2.5–3% isoflurane-oxygen and placed in a stereotactic frame secured by ear and nose bars adapted specifically for the rat. 100 μL of 1% lidocaïne was applied immediately on the skin incision. To achieve lesioning of the nigrostriatal pathway, 1.5 μL of 6-OHDA (2 μg/μL in 0.1% ascorbic acid dissolved in saline to prevent heat and light exposure) was injected into the right medial forebrain bundle at 2 injection sites (3 μL total injected volume): with coordinates related to Bregma as follows: first site, AP = −2.0 mm; ML = −1.0 mm; DV = −8.0 mm; second site, AP = −2.6 mm; ML = −1.8 mm; DV = −8.2 mm. The Sham group received saline injection and corresponded to historical unpublished sham data used as reference in behavioral analyses, as sham performance has been found to be highly consistent across experiments. Rats received a buprenorphine injection in the evening after stereotactic surgery and the following morning. The surgery was conducted under strict aseptic conditions, with the animal’s body temperature maintained using a heating pad. Based on validation from previous experiments using the same injection parameters, no postmortem verification was performed in the present study.

#### 2.3.3 DCE-δ3 intranasal treatment

The 6-OHDA group was randomly divided into two groups, one of which received DCE-δ3 and the other the vehicle. One group received 4 mg/day of DCE-δ3, given by the IN route corresponding to 13.33 mg/kg/day for a rat of 300 g. 25 μL of the emulsion was placed in each nostril twice a day at fixed 7-h intervals for a total daily volume of 100 μL per rat. The DCE-δ3 treatment started 1 day before the 6-OHDA intoxication. On the day of stereotactic surgery, rats received only one IN treatment immediately after the end of surgery. During the 5 first days after surgery, the administration was performed under isoflurane anesthesia in order to protect the rats’ wounds during the restraint required for dosing. The day of behavioral tests, rats only received the evening treatment. The total duration of the treatment was 5 weeks. A group of 6-OHDA-intoxicated animals received the equivalent volume of vehicle.

#### 2.3.4 Induced rotation tests

Three weeks after the injection of 6-OHDA, animals were tested for their turning behavior following a challenge with amphetamine and apomorphine. The amphetamine and apomorphine tests were conducted on separate days. Rats received an intraperitoneal injection of D-amphetamine (2.5 mg/kg) or subcutaneous apomorphine (0.1 mg/kg). 15 min after injection, their activity was recorded for a 15-min period in a round shape open field arena (60 cm diameter). The number of 360-degree ipsiversive or contraversive rotations was recorded manually by an observer who was blinded to the treatment groups. The arena was cleaned with 70% ethanol between each trial of consecutive animals. For reference, historical data from our unpublished observations of sham-operated ratswere used.

#### 2.3.5 Beam walking test

Four weeks after the 6-OHDA injection, sensorimotor coordination was assessed by placing each rat on a 220 cm-long, 2 cm-wide flat rectangular wooden beam, divided into four 50-cm segments, elevated 80 cm above the floor level, and which was in contact with the home cage at one end. All rats were trained according to the following protocol. On the first session (day 1), the rats were placed on the beam, 50 cm away from the home cage on five consecutive occasions. On the next session (day 2), the rats are placed 50, 100, 150 and 200 cm away from the home cage, successively, with only one run allowed for each distance. On the third session (day 3), the rats were placed twice 100 cm away and then twice 200 cm away from the home cage. On the fourth session (day 4), the rats were placed 200 cm away for three consecutive runs. On the next day, all rats were tested for three consecutive trials as in the fourth session, and their performances were rated.

For each virtual 50-cm segment of the beam, the experimenter rated the locomotor behavior (walking score) as follows:

- a score of 1 per segment when the rat crossed the segment with all paws on the upper surface of the beam,
- a score of 0 per segment on which the rat slipped, placed its toes on the side surface of the beam or fell from the beam.
- The overall score was calculated by adding the scores of the three trials (maximum score: 12, i.e., 4 for each trial), and the interval between each trial was 5 s.
- Rats that did not move within 120 s after initiation of the test were considered to have delayed motor initiative (limb akinesia). The number of rats with limb akinesia was counted.

The number of segments crossed within 120 s was counted and the crossing time was recorded. For reference, historical data from our unpublished observations of sham-operated rats were used.

#### 2.3.6 Brain, plasma sampling

At the end of the experiment, rats are treated with a last dose of DCE or its vehicle then after 2 h blood and brain were collected from each rat. Briefly, rats were anaesthetized with isoflurane. Then, blood was collected from each animal by cardiac puncture to prepare plasma. Blood was withdrawn slowly from the animal on heparinized syringe. Then, it was centrifuged at 4°C at 1300 g for 10 min at 4°C and the plasma was transferred into clean pre-labelled tubes. Two plasma samples per rat were prepared. In 6 rats per group, the whole brain (including the cerebellum) was extracted and divided as right and left hemispheres which were rapidly snap frozen. In the remaining rats, the brain was dissected as cortex, striatum, hippocampus and cerebellum. For reference, age-matched naïve rats were used.

### 2.4 Quantification of DCE-δ3, creatine-δ3, and creatinine-δ3 in biological media

#### 2.4.1 Quantification in plasma

20 μL of plasma was diluted with 60 μL of acetonitrile and 5% acetic acid. Internal standard (unlabeled DCE, Cr-δ6 and creatinine-δ6) was added (5 μL) to all samples and calibration standards. After vortex mixing, the samples were centrifuged at 20,000 g for 10 min at 4°C. 50 μL of the supernatant was then diluted with 50 μL of acetonitrile. The samples were centrifuged at 20,000 g for 10 min at 4°C before injecting 5 μL into the LC–MS/MS system (Xevo TQS and Acquity I-Class, from Waters, Guyancourt, France). The column was an Aquity UPLC BEH Amide 100 × 2.1.0 mm, 1.7 μm and the mobile phase was delivered at 0.6 mL/min and consisted of a gradient of 10 mM ammonium acetate in water with 1% acetic acid (A) and acetonitrile with 1% acetic acid (B) as follows: from 0 to 0.5 min, 5% A; from 0.5 to 2 min, a linear gradient to 60% A; rapidly increased at 2.01 to 80% A; maintained until 2.5 min; then returned to starting conditions at 2.51 min; and equilibration until 5 min. The autosampler was kept at 4°C or less than 10°C and the oven at 60°C. Retention times of DCE-δ3, Cr-δ3 and creatinine-δ3 (Crn-δ3) were 1.4, 2.0 and 1.6 min, respectively. Detection was done by tandem mass spectrometry in positive electrospray mode. Spray voltage was 3.0 kV and sheath and auxiliary gas pressures were 50 and 20 (arbitrary units), respectively. Tube lens and collision energy values were optimized for DCE compounds. The transitions were based on the m + 1 ion for each compound, ie. 303 for DCE, 135 for Cr and 117 for Crn. Fragment ions were 93, 93 and 47 for DCE-δ3, Cr-δ3 and Crn-δ3, respectively. The limit of detection (LOQ) for DCE-δ3, Cr-δ3 and Crn-δ3 was 0.8, 2.6 and 1.9 ng/mL, respectively.

#### 2.4.2 Quantification in brain tissues

The method was similar to the previous one used for plasma samples, except that sample preparation was carried out as follows: 200–300 mg of tissue was mixed in 3 volumes of water. 50 μL of the brain homogenate was diluted with 200 μL of a mixture of acetonitrile and acetic acid. The process was identical for the plasma samples afterwards. The LOQ was 1.2, 8 and 3 ng/g of tissue for DCE-δ3, Cr-δ3 and Crn-δ3, respectively.

### 2.5 Quantification of dopamine, serotonin, and nicotinamide adenine dinucleotide in brain samples

The levels of dopamine (DA) and serotonin (5-HT) and nicotinamide adenine dinucleotide (NAD) were quantified using high-performance liquid chromatography (HPLC) on crushed striatal samples as described previously (Bernal-Meléndez et al., 2021; Pittaras et al., 2018). The striatum was weight (*n* = 5–6 per group) and was crushed 400 μL of 0.2 M perchloric acid and centrifuged at 22,000 g for 20 min at 4°C. The supernatants were collected and filtered through a 10 kDa membrane (Nanosep, Pall) by centrifugation at 7000 g (30 min). Then, a 20 μL aliquot of each sample was analyzed for 5-HT by fluorometric detection (Kema). The amounts of dopamine, 5-HT and NAD were measured by electrochemical detection on a serial array of coulometric flow-through graphite electrodes (CoulArray, ESA). Analysis, data reduction, and peak identification were fully automated. Results were expressed as femtomoles/milligram of fresh tissue.

### 2.6 Western blot

Western blotting was used to detect the abundance of neurofilaments (Nf-L, Nf-M, Nf-H) and BDNF/pro-BDNF. Striatal tissues were homogenized in freshly prepared lysis buffer containing 20 mM Trizma-Base, 150 mM NaCl (pH 7.4) (Sigma-Aldrich, Saint-Quentin Fallavier, France), 1% Triton X-100, 4% complete protease inhibitor cocktail (cOmplete, Roche) and a 20% mix of anti-phosphatase inhibitors (ammonium molybdate, sodium glycerophosphate, sodium fluoride, sodium pyrophosphate, sodium orthovanadate) using a Precellys Evolution tissue homogenizer. The samples were then centrifuged at 2500 g for 15 min followed by 10,000 g for 20 min to obtain lysates for electrophoresis. The proteins (40 μg) and protein standards were mixed with Laemmli buffer and loaded on 4–15% Criterion TGX Stain-Free protein gels in 1 × TGS running buffer (all from Bio-Rad, Marnes-la-Coquette, France) and transferred to a 0.2 μm PVDF membrane with the Trans-Blot Turbo RTA Midi Transfer Kit (Bio-Rad, Marnes-la-Coquette, France). The membranes were blocked for 30 min in 5% low-fat milk in TBS-0.1% Tween 20 at room temperature. The blots were probed with specific primary antibodies overnight at 4°C followed by horseradish peroxidase (HRP) secondary antibodies diluted 1:10000 in 5% low-fat milk in TBS-0.1% Tween 20 at room temperature. For protein detection, membranes were exposed to the Immobilon Crescendo or Forte Western HRP substrate (Millipore) and exposed with a ChemiDoc Touch Imaging System (Bio-Rad, Marnes-la-Coquette, France). The band density was quantified with Image Lab software (Bio-Rad, Marnes-la-Coquette, France). The following antibodies were used at the indicated dilutions: anti-BDNF that recognize both BDNF (14 kDa) and pro-BDNF (32 kDa) (1:500, Abcam, ab108319), anti-Nf-L (68 kDa; 1/500, Abcam, ab7255), anti-Nf200 that recognize both Nf-M (160 kDa) and Nf-H (200 kDa) (1:500, Sigma-Aldrich, N0142), anti-GFAP (1:1000, Cell Signaling, 80,788), anti-GAPDH (36 kDa;1:2500, Sigma-Aldrich, G8795) or anti-*α*-tubulin (50 kDa;1:2000, Sigma-Aldrich, T6199) were used for normalization.

### 2.7 Quantification of neurofilaments in plasma

Nf-L concentration was determined using the commercial NF-Lv2 kit (ref 104,073, Quanterix, USA) based on ultrasensitive Simoa® technology. All samples were diluted with the provided dilution buffer to minimize matrix effects. Quality controls with low Nf-L concentration (QC 1 with mean concentration of 13.3 pg./mL) and QC high Nf-L known concentration (QC 2 with mean concentration of 519.9 pg./mL) were provided in the kits. We observed a low inter-assay variation for QC 1 and QC 2 with a coefficient of variation (CV) of 4.2 and 0.7%, respectively. All experiments were performed with a single batch of reagents. These determinations were conducted at the clinical proteomic platform, IRMB, Montpellier, France (Profs S. Lehmann & C. Hirtz).

### 2.8 Statistical analysis

Analyses were performed using the Prism 10.3 program (GraphPad Software, Inc., San Diego CA). After homogeneity of variance confirmation by the Bartlett test, statistical comparisons of several groups in motor behavior experiments were performed by one-way ANOVA followed by the Tukey *post hoc* test. Dopamine, NAD and 5HT levels in the DCE-δ3-treated and vehicle-treated 6-OHDA groups were compared by the Mann–Whitney test. Western blot analysis was compared by the Kruskal-Wallis test followed by Dunn’s uncorrected post hoc test.

## 3 Results

### 3.1 Intranasal DCE-δ3 significantly ameliorated 6-OHDA-induced sensorimotor impairments

**Figure 2:**
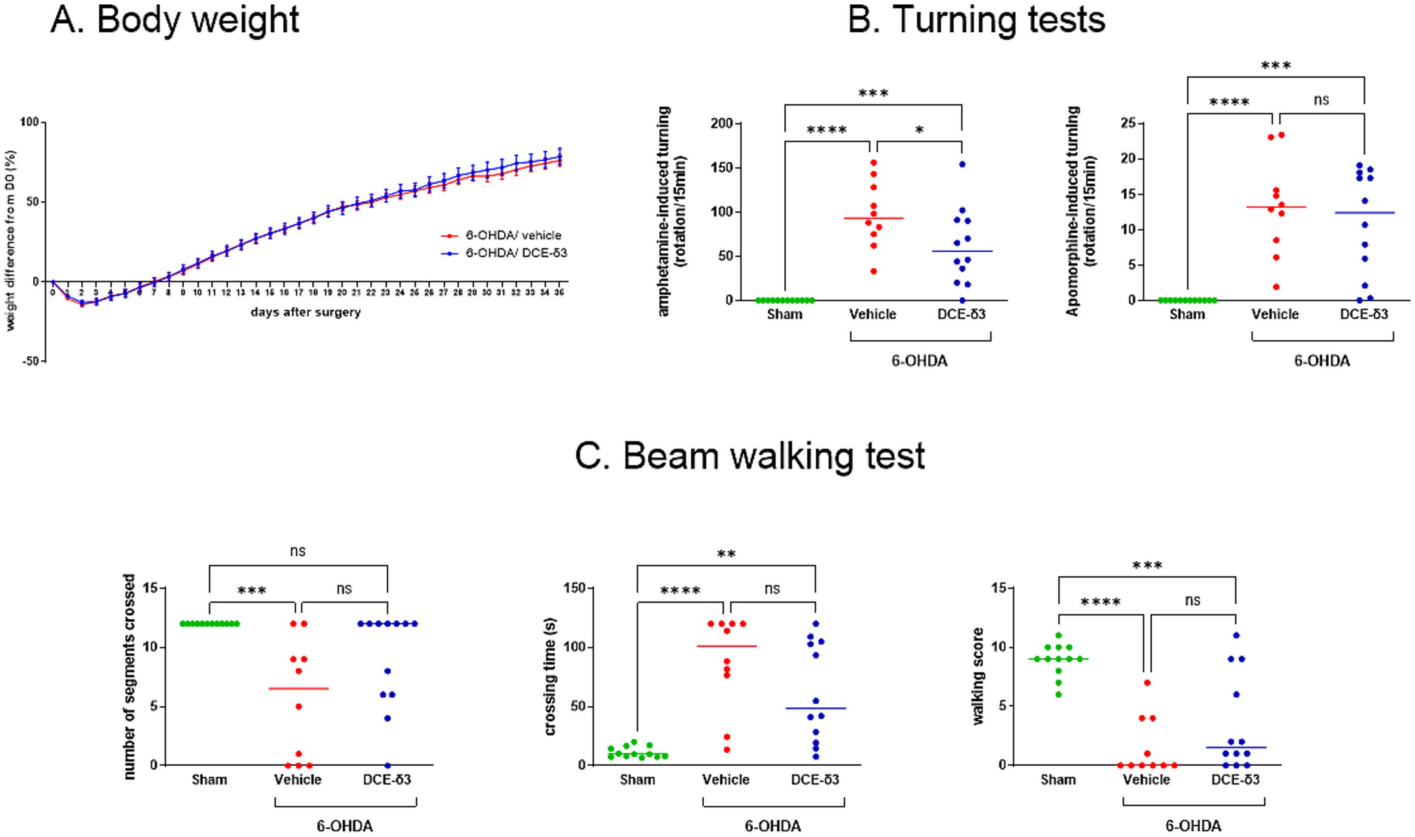
Amphetamine-induced rotational behaviors and beam walking tests in 6-OHDA-intoxicated rats after intranasal treatment with DCE-δ3. **(A)** Body weight monitoring in 6-OHDA-intoxicated rats after stereotactic administration up to the end of IN DCE-δ3 treatment for 5 weeks. Weight is expressed as a percentage from the day of 6-OHDA injection (D0) and is shown as the mean in each group ± SEM, *n* = 10 to 12 rats **(B)** Amphetamine-and apomorphine-induced turning test in 6-OHDA rats after 4 weeks of intranasal treatment with DCE-δ3. Results are expressed as the number of rotations per 15 min after challenge with amphetamine or apomorphine. Tests were performed after 3 weeks of treatment **(C)** Beam walking test in 6-OHDA rats after 4 weeks of intranasal treatment with DCE-δ3. Performance evaluated by number of segments crossed, crossing time in seconds, and walking score. Tests were performed after 4 weeks of treatment. Each data point represents one animal, with a median of *n* = 10 to 12 rats. Statistical comparison between sham rats, 6-OHDA-intoxicated rats treated with the vehicle, and 6-OHDA-intoxicated rats treated with CBT101-δ3 for a month was performed by one-way ANOVA followed by Tukey’s *post hoc* test. * *p* < 0.05; ** *p* < 0.01; ***p < 0.001; ****p < 0.0001.

As depicted in Figure 2A, all 6-OHDA-intoxicated groups experienced weight loss in the initial days following surgery. Nonetheless, by the seventh day post-surgery, the animals had regained their body weight, showing no significant difference compared to day 0 (*p* > 0.05). No differences were found between the vehicle-and DCE-δ3-treated 6-OHDA-intoxicated groups.

Using a comprehensive set of motor behavior tests, our results showed that IN DCE-δ3 treatment resulted in significant functional recovery in the 6-OHDA-intoxicated rats. First, 3 weeks after the injection of 6-OHDA, animals were tested for their turning behavior following a challenge with amphetamine or apomorphine. Upon amphetamine the 6-OHDA-intoxicated rats treated with the vehicle showed significantly increased ipsiversive rotations (towards the lesion side), whereas apomorphine challenge induced contraversive rotations (away from the lesion side) (*p* < 0.05) (Figure 2B). IN DCE-δ3 treatment for 3 weeks significantly reduced rotations induced by amphetamine injection as compared to the 6-OHDA/vehicle rats (*p* < 0.05). The DCE-δ3-treated group exhibited no improvement in the postsynaptic challenge with apomorphine.

To assess sensorimotor coordination, the beam walking test was performed 4 weeks after the 6-OHDA administration. The 6-OHDA/vehicle intoxicated group spent significantly more time crossing the beam, crossed fewer segments, and had a lower mean score in this test compared to the sham group (Figure 2C). A comparison of walking patterns among the different groups revealed that IN DCE-δ3 treatment for 4 weeks increased the walking score, especially regarding the percentage of animals reaching a score higher than zero (40% in the 6-OHDA/vehicle group versus 75% in the 6-OHDA/DCE-δ3 group). Additionally, IN DCE-δ3 treatment decreased the time that the rats needed to cross the beam and increased the number of crossed segments (Figure 2C). We noted an increased number of animals crossing all segments of the beam in the 6-OHDA/DCE-δ3 group compared to the 6-OHDA/vehicle group (20% in the 6-OHDA/vehicle group versus 58% in the 6-OHDA/DCE-δ3 group), and a decreased number of animals not moving during the test (40% in the 6-OHDA/vehicle group versus 8% in the 6-OHDA/DCE-δ3 group). Altogether, these results indicate improved motricity in the 6-OHDA group of rats treated with DCE-δ3.

### 3.2 Creatine concentration in brain and plasma

Plasma and brain samples of 6-OHDA-intoxicated rats treated with DCE-δ3 were collected after 5 weeks of IN DCE-δ3 treatment. Stable isotope-labeled DCE (DCE-δ3) was administered and used to differentiate metabolites derived from the treatment from endogenous compounds, as the labeling was done on the Cr side of the prodrug molecule (Supplementary Figure 1). DCE-δ3, Cr-δ3, and creatinine-δ3 (Crn-δ3) were quantified using LC–MS/MS. These compounds were found in both brain hemispheres (right being the 6-OHDA-intoxicated hemisphere), without notable differences (Table 1). DCE-δ3 was detected at very low levels in the brain, while Cr-δ3, the active metabolite, was the predominant species in the brain compared to Crn-δ3.

**Table 1:** DCE-δ3, creatine-δ3 and creatinine-δ3 concentrations in brain and plasma of 6-OHDA rats after 5 weeks of intranasal treatment with DCE-δ3.

Samples		DCE-δ3	Creatine-δ3	Creatinine-δ3
Brain (ng/g)	Right hemisphere	2.63 ± 1.55	253 ± 104	8.35 ± 2.27
	Left hemisphere	2.22 ± 1.18	302 ± 101	8.91 ± 1.82
Plasma (ng/mL)		BLOQ	21.48 ± 15.32	92.91 ± 32.05

Quantification by LC–MS/MS of DCE-δ3, creatine-δ3 and creatinine-δ3 in the brain (right and left hemisphere separately) and plasma collected 2 h after the last administration in 6-OHDA-intoxicated rats treated for 5 weeks with 13.3 mg/kg/day of intranasal DCE. Results presented as a mean ± SD for 12 animals. BLOQ = below the limit of quantification. For plasma samples, the limit of quantification (LOQ) for DCE-δ3, Cr-δ3 and Crn-δ3 was 0.8, 2.6 and 1.9 ng/mL, respectively. For brain samples, the LOQ was 1.2, 8 and 3 ng/g of tissue for DCE-δ3, Cr-δ3 and Crn-δ3, respectively.

As expected, DCE-δ3 was not detected in plasma because it is rapidly degraded into Crn-δ3 in biological fluids. Therefore, plasma levels of Cr-δ3 and Crn-δ3 reflect the systemic distribution of DCE-δ3, confirming that a portion of the administered dose is absorbed and distributed systemically. Consistent with previous studies, Crn-δ3 was the dominant species in plasma.

### 3.3 Intranasal DCE significantly increase dopamine levels in the striatum after 6-OHDA intoxication

**Figure 3:**
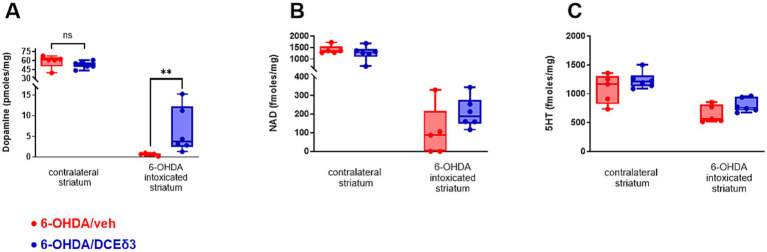
Concentration of dopamine, nicotinamide adenine dinucleotide and serotonin in right striatum of 6-OHDA-intoxicated rats after intranasal treatment with DCE-δ3. Dopamine **(A)**, nicotinamide adenine dinucleotide (NAD) **(B)** and serotonin (5HT) **(C)** measured by HPLC in 6-OHDA-intoxicated rat striatum with or without IN DCE-δ3 treatment for 5 weeks. Results presented as concentrations in the 6-OHDA-intoxicated striatum (right hemisphere) and ratio of concentration in the right striatum injected with 6-OHDA over the left. Statistical comparison was performed by the Mann–Whitney test. **p < 0.01. *n* = 5–6 animals.

In half of the animals in both the 6-OHDA/vehicle and 6-OHDA/DCE-δ3 groups, the striatum was isolated to quantify dopamine, NAD, and serotonin (5-HT) (Figure 3). Results are presented as concentrations of dopamine, NAD, and 5-HT in the 6-OHDA-intoxicated hemisphere (right) and the non-intoxicated hemisphere (left) in both 6-OHDA/vehicle and 6-OHDA/DCE-δ3 groups (Figure 3).

In the striatum intoxicated by 6-OHDA, dopamine levels were significantly higher in animals treated with DCE-δ3 compared to those treated with the vehicle (Figure 3A). The mean concentration of dopamine in the right lesioned hemisphere was 0.65 pmoles/mg in the 6-OHDA/vehicle group versus 6.35 pmoles/mg in the 6-OHDA/DCE-δ3 group, thus corresponding to a 10-fold increase with treatment. The dopamine level in the striatum correlated highly statistically with all observed sensorimotor performances (correlation computed using the Spearman nonparametric correlation, Supplementary Figure 2). NAD and serotonin (5-HT) exhibited a similar pattern of modulation, with a slight tendency toward higher levels after treatment with DCE, but without a statistically significant effect (Figures 3B,C).

### 3.4 Intranasal DCE-δ3 treatment modulates neurofilament expression in the striatum and plasma

**Figure 4:**
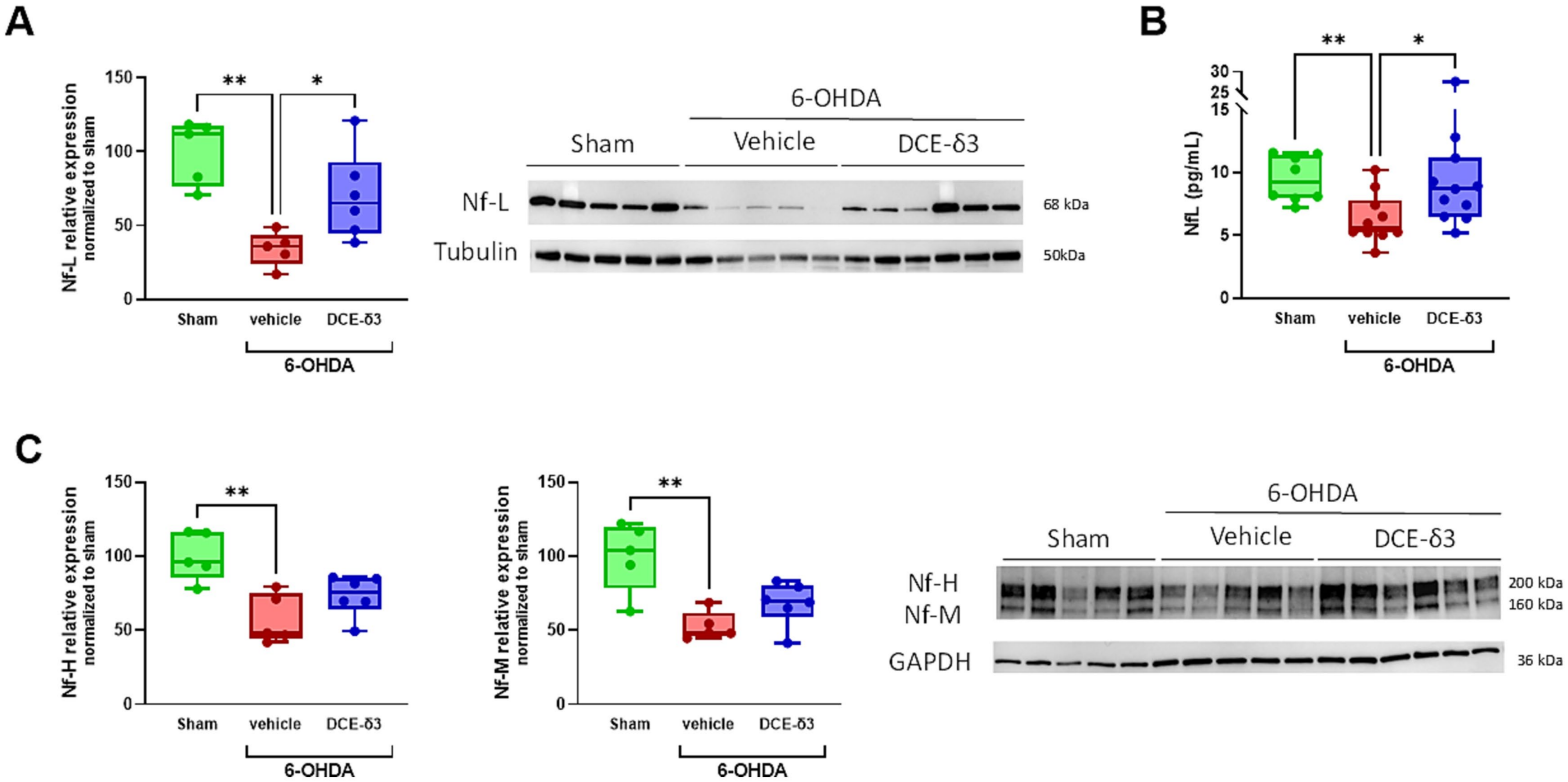
Modulation of neurofilament expression in the striatum and plasma of 6-OHDA-intoxicated rats after 5 weeks of intranasal treatment with DCE-δ3. Western blot analysis of proteins levels Nf-L **(A)** and Nf-M and Nf-H **(C)** in the striatum of the sham, 6-OHDA/vehicle and 6-OHDA/DCE-δ3 groups, 5 weeks after the 6-OHDA intoxication and the intranasal treatment. Graphs showing densitometric analysis of intensity of immunoblots. Values normalized to the sham group set at 100%. Circulating Nf-L were measured in plasma **(B)** by the Simoa^R^ Technology (from Quanterix). Results expressed in pg/mL of plasma. Statistical comparison was performed by the Kruskal-Wallis test followed by Dunn’s uncorrected post hoc test. * *p* < 0.05; **p < 0.01. *n* = 5–6 animals for western blotting and *n* = 10–12 for circulating NF-l quantification in plasma.

To characterize impact on neurons, neurofilament light chains (Nf-L), medium chains (Nf-M) and heavy chains (Nf-H) were quantified by western blot in the striatum (Figure 4). Nf-L levels in the striatum decreased by 66% in the 6-OHDA/vehicle group compared to the sham group (Figure 4A). The 5-week IN DCE-δ3 treatment significantly moderated this reduction due to the 6-OHDA intoxication, with a decrease limited to 30% compared to the sham group.

In an intent to find circulating biomarkers easily accessible for therapeutic efficacy monitoring, we also quantified Nf-L in plasma samples. The modulation pattern in plasma was consistent with findings in the striatum (Figure 4B), indicating that circulating Nf-L levels reflect those in the central nervous system. In addition, Nf-M and Nf-H levels also showed a significant decrease in the striatum following 6-OHDA intoxication (decreased by 47.2 and 42.5% compared to the sham group for Nf-M and Nf-H, respectively; Figure 4C). The IN DCE-δ3 treatment tended to increase expression of both Nf-M and Nf-H compared to the 6-OHDA/vehicle group, albeit without statistical significance.

### 3.5 Intranasal DCE-δ3 treatment modulates striatal pro-BDNF/BDNF balance

**Figure 5:**
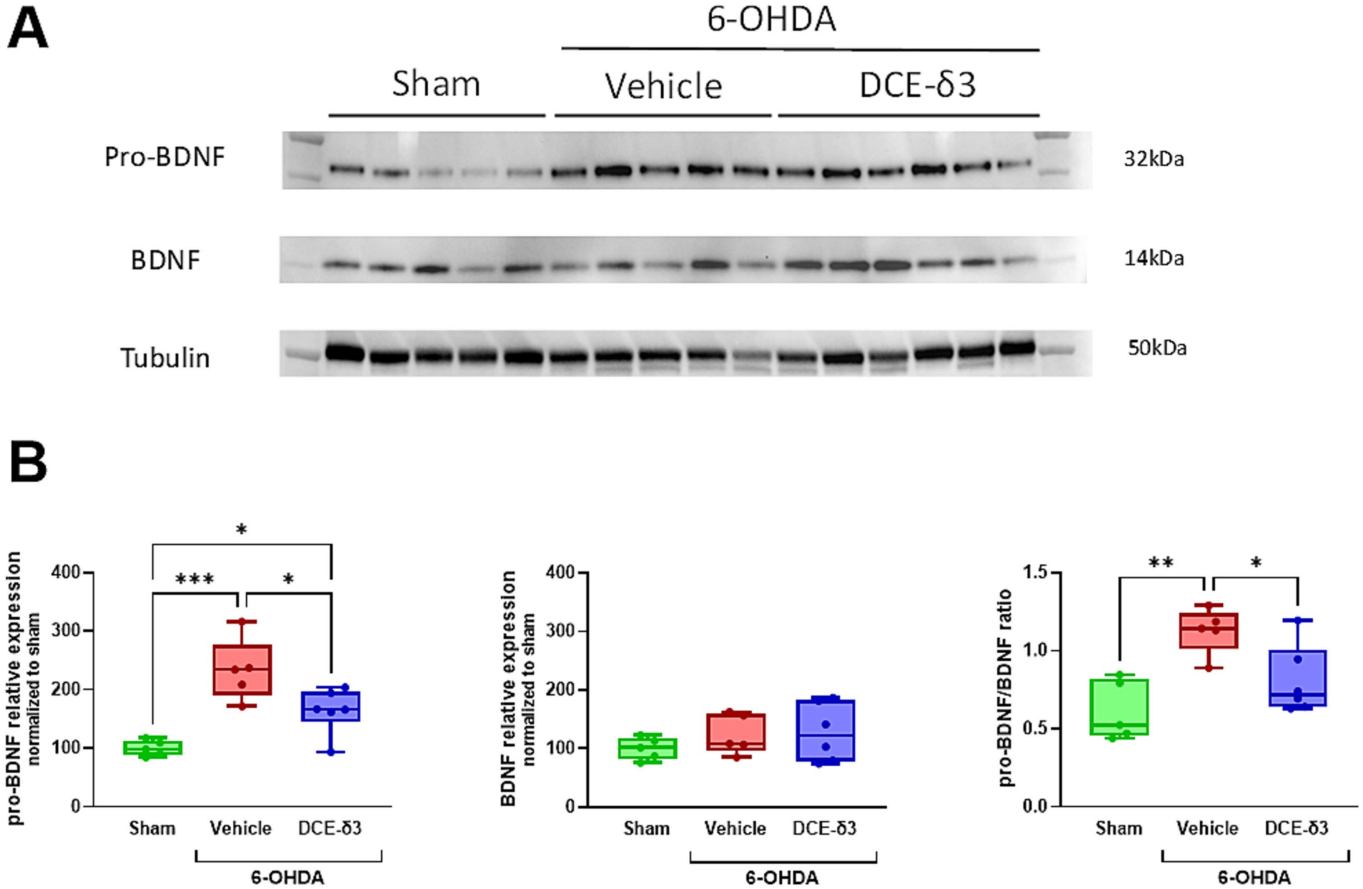
Modulation of pro-BDNF/BDNF balance in the striatum of 6-OHDA-intoxicated rats after 5 weeks of intranasal treatment with DCE-δ3. **(A)** Representative western immunoblot of pro-BDNF and BDNF in the striatum of the sham, 6-OHDA/vehicle and 6-OHDA/DCE-δ3 groups, 5 weeks after the 6-OHDA intoxication and the intranasal treatment. **(B)** Graphs showing densitometric analysis of the intensity of immunoblots. Values were normalized to the sham group set at 100%. Statistical comparison was performed by the Kruskal-Wallis test followed by Dunn’s uncorrected post hoc test. * *p* < 0.05; **p < 0.01. *n* = 5–6 animals.

In an attempt to shed light on the mechanism accounting for the neuroprotective effect of DCE-δ3, we investigated the impact of the treatment on striatal pro-BDNF/BDNF balance (Figure 5). The expression of pro-BDNF was 2.3 times higher in the 6-OHDA/vehicle group compared to the sham group (*p* < 0.05; Figure 5B). The 5-week IN DCE-δ3 treatment significantly reduced pro-BDNF expression in the striatum of 6-OHDA-intoxicated animals, with the level corresponding to 1.6 times that of the sham group. No significant differences were observed in mature BDNF expression between any of the experimental groups (*p* > 0.5, Figure 5B). To ascertain whether the pro-BDNF/BDNF balance was impacted by 6-OHDA intoxication and IN DCE-δ3 treatment, ratios of pro-BDNF to mature BDNF were calculated. The ratio of pro-BDNF/BDNF significantly increased in the 6-OHDA/vehicle group compared to the sham group, and was significantly lowered after IN DCE-δ3 treatment.

## 4 Discussion

This study examined the cerebral effects of intranasally administered DCE labeled with three stable isotopes (e.g., DCE-δ3) in rats with unilateral MFB 6-OHDA lesions. By using a stable labeled molecule, creatine-δ3 and creatinine-δ3 originating from metabolization of DCE-δ3 can be monitored by differentiating them from endogenous, thus unlabeled, creatine and creatinine. Rats intoxicated with 6-OHDA showed motor impairments in beam walking tests and an abnormal rotational behavior in response to apomorphine and amphetamine challenge. Additionally, these rats experienced dopamine levels drastic decrease, reduced neurofilament expression and imbalance in the pro-BDNF/BDNF ratio in the striatum. IN DCE effectively mitigated these motor symptoms and related biochemical modifications. In addition, modulation of neurofilaments was investigated in the striatum and Nf-L was identified as a potential biomarker of DCE efficacy in plasma. The mechanism of DCE’s positive impact on neurons could be related in part to modulation of pro-BDNF levels.

Toxin administration in specific brain regions of rodents, such as the medial forebrain bundle, has been widely reported to cause degeneration of dopaminergic neurons and to mimic certain clinical aspects of mitochondrial diseases such as PD (Khan et al., 2023; Thirugnanam and Santhakumar, 2022; Casanova et al., 2022). 6-OHDA is a dopamine analogue that selectively causes degeneration of dopaminergic neurons in the substantia nigra via several mechanisms, including the production of free radicals and direct inhibition of mitochondrial complex I in the respiratory chain (Khan et al., 2023; Thirugnanam and Santhakumar, 2022; Casanova et al., 2022). The substantia nigra sends dense dopaminergic projections to the striatum, the major input nucleus of the basal ganglia. Neuronal projections from the substantia nigra are also sent to other brain regions, leading to widespread network adaptations with their loss in PD (Calabresi et al., 2013; Galvan et al., 2015). The substantia nigra contains the neuronal cell bodies. However, the striatum contains dopaminergic neurons terminals that release dopamine. Consequently, intoxication in the substantia nigra leads to retrograde degeneration (from the cell body toward the terminals) and changes in the striatum are directly linked to functional loss (Chuhma et al., 2023). Therefore, measuring the impact in the striatum allows for assessment of the effective loss of functional dopaminergic transmission. In addition, the striatum is more accessible for biochemical measurements. The 6-OHDA-induced model replicates several cellular processes observed in PD, making it suitable for studying the molecular basis of cytotoxicity, oxidative stress, and neuronal death. One limitation of the 6-OHDA toxin model is that it does not mimic the progressive loss of dopaminergic neurons and lacks the Lewy-related pathology seen in PD. Therapeutics currently used to treat other neurodegenerative diseases also lead to positive outcomes in the 6-OHDA lesion model. As an example, riluzole, which is used to treat ALS patients, attenuates glutamatergic overactivity (Gilgun-Sherki et al., 2003; Barnéoud et al., 1996).

Rotation tests are classical tests used in rodent neurotoxin PD models with ipsilateral lesions. Amphetamine or apomorphine is used to investigate the extent of loss of dopaminergic cells induced by 6-OHDA (Bjorklund and Dunnett, 2019). When amphetamine or apomorphine is administered to unilaterally 6-OHDA-intoxicated rats, dopamine is released in greater amounts in the intact striatum than in the intoxicated side, thus producing an asymmetric motor activation of the right and left sides of the body. The result is an intensive ipsilateral rotational behavior, which correlates with the extent of dopaminergic denervation. Amphetamine acts presynaptically to stimulate dopamine release and/or block dopamine reuptake. In the absence of corresponding dopamine stimulation on the intoxicated side, the higher concentration of released dopamine in the intact striatum results in rotation in the direction ipsilateral to the lesion. IN DCE treatment significantly lowers the number of rotations induced by amphetamine. These results would suggest neuroprotection and/or enhancement of neurogenesis of dopaminergic neurons. Investigations about those two hypotheses remain to be done. Dopamine receptor agonists, such as apomorphine, act postsynaptically and because of hyperstimulation of supersensitive dopamine receptors in the denervated striatum induce rotation in the opposite contralateral direction. Animals treated with IN DCE exhibit no significant improvement in the postsynaptic challenge with apomorphine compared with the vehicle-treated group. These results suggest that there is no postsynaptic effect and that the remaining neurons are not hyperexcitable. This observation was further supported by the fact that DCE IN treatment increases dopamine in the right lesioned striatum of the 6-OHDA-intoxicated DCE treated group compared to the vehicle treated group. Modulation of 5-HT and NAD have a consistent pattern without statistical significance. However, further histological analysis will be needed to further investigate the impact of DCE treatment on neurons of the nigrostriatal pathway.

During the beam walking test, the number of segments crossed was improved (49% increase in the median) as was the crossing time (52% decrease in the median) in the DCE-treated group compared to the vehicle-treated group. In addition, the number of animals with a walking score above zero was increased in the DCE-treated group compared to the placebo-treated group. This suggests improvement in the motor functions observed in the 6-OHDA model. To confirm the motor function improvement, we measured the dopamine levels in the striatum. The tenfold increase in dopamine concentrations in the striatum of intoxicated rats confirmed the positive effect of the treatment. NAD depletion is involved in PD pathophysiology because it is implicated in redox reactions and reduced levels may cause mitochondrial dysfunction and neurodegeneration (Shan et al., 2019). Measurements also showed a dramatic decrease in NAD levels in the 6-OHDA-lesioned hemisphere and a positive impact of the IN DCE treatment, despite the lack of statistical significance. The lack of significance is likely due to the small number of animals in which these measurements could be carried out, combined with the low concentrations of NAD in the analyzed samples. Several studies have demonstrated that the serotoninergic system is also altered in PD, suggesting that dopaminergic neurons are not the only neuronal subtypes impacted (Boi et al., 2024). We demonstrated that 5-HT is indeed decreased in the 6-OHDA-lesioned striatum.

After 5 weeks of treatment, we examined the brain distribution of exogenous Cr-δ3 (labeled with stable isotope) in this 6-OHDA rat model. The data obtained once again confirmed previous observations in non-human primates and in a mouse model of Cr transporter deficiency (Disdier et al., 2025), confirming that the dual strategy of combining a Cr prodrug with nasal administration effectively delivers Cr to the neurons of the striatum, despite the almost complete lack of expression of the SLC6A8 transporter in these neurons (Disdier et al., 2025). The analysis of brain distribution could not be performed at the regional or cellular level in this study. Ergogenic effect of Cr is well documented (Forbes et al., 2022; Roschel et al., 2021). We hypothesized that this enhanced energy supply could be beneficial in Parkinson’s disease (PD) and other conditions characterized by energy deficits and mitochondrial dysfunction. In a previous study, we demonstrated that intranasal treatment with DCE led to increased ATP levels in the striatum (Ullio-Gamboa et al., 2019). However, in the current study, it would have been valuable to analyze ATP levels specifically in the striatum region.

Neurofilaments are components of a family of intermediate filament proteins localized in the axonal cytoplasm of neurons. In mature myelinated axons, neurofilaments are the most abundant proteins and play a crucial role in maintaining axonal structure. The presence of neurofilaments in cerebrospinal fluid (CSF) and, eventually, in the blood is thought to result from the normal turnover of these proteins. Nf-Ls are also released into the extracellular fluid when axons are injured or degenerate and can be measured in CSF and blood, making it a strong biomarker candidate for various neuropathological diseases, including PD (Buhmann et al., 2023; Park et al., 2024). Consequently, Neurofilaments could be used as a markers of neuronal injury in tissue but also in circulating biological fluids. We hypothesized that such biomarkers could be used for therapeutic efficacy monitoring. In this study, we report decreased levels of neurofilaments in the striatum and reduced Nf-L levels in the serum of 6-OHDA-intoxicated rats. A similar decrease in Nf-H was previously reported by Li et al. in the striatum and substantia nigra after 6-OHDA intoxication in Wistar rats (Li et al., 2019). Fuller et al. described increased Nf-H expression during ongoing nigrostriatal degeneration (3 days post lesioning), but followed by a reduction in its expression in the fully denervated striatum (7 and 14 days post lesioning) (Fuller et al., 2014). Conversely, an increase in serum Nf-L levels was observed by Kasanga et al. 4 weeks after 6-OHDA intoxication in Sprague–Dawley rats (Kasanga et al., 2024). In addition, Kumari et al. reported an increase of Nf-L measured by immunostaining in the brains of Sprague–Dawley rats 1 month after intoxication with 250 μg 6-OHDA (Kumari et al., 2021).

This discrepancy may be attributed to differences in experimental methodologies. The extent of dopaminergic denervation depends on factors such as the type and dose of the toxins, the injection site, whether the lesions are unilateral or bilateral, and the age and species of the animals. We hypothesized that the low dose of 6-OHDA used in this study (3 μg unilaterally) may have initially caused an increase in circulating Nf-L due to neuronal damage, followed by a subsequent decline associated with the reduced number of surviving neurons. Our findings suggest that 6-OHDA-induced axonal atrophy led to decreased Nf-L levels in both the striatum - the lesioned brain region - and the blood circulation 5 weeks post-intoxication. The restoration of neurofilament levels in both the central nervous system and plasma following 5 weeks of IN DCE treatment suggests that axons were either preserved or that axogenesis was stimulated by DCE. In the future, it will be interesting to investigate the kinetics of the modulation of circulating Nf-L after intoxication in preclinical models of PD.

In previous studies, a positive impact of DCE treatment on markers such as BDNF has been highlighted (Ullio-Gamboa et al., 2019; Mabondzo et al., 2023; Disdier et al., 2025). BDNF is the most widely distributed and most abundant growth factor in the central nervous system. The BDNF signaling pathway is key for synaptic plasticity and transmission, through regulation of several mitochondrial processes such as mitochondrial bioenergetics, biogenesis, and dynamics (Bathina and Das, 2015).

Therefore, we hypothesize that IN DCE treatment alleviated motor dysfunction in rats with PD by upregulating BDNF in the brain, which in turn would lead to a series of neuroprotective effects, including neurogenesis, synaptic plasticity, axonal and dendritic growth, and long-term potentiation of neurons. Mature BDNF is generated from pro-BDNF by proteolytic cleavage. BDNF binds to its receptor, tyrosine kinase receptor B (TrkB), to activate a signaling cascade, leading to neuroprotective effects. Pro-BDNF binds to another receptor, the p75 neurotrophin receptor (p75NTR), to induce apoptosis. Consequently, pro-BDNF and mature BDNF have opposite effects and modulation in pro-BDNF/BDNF balance can have a major impact on neuronal function (Ali et al., 2024). Several studies reported that BDNF support proper striatum functions and the BDNF/TrkB pathway is important in motor coordination including in pathological context such as parkinsonism (Baydyuk and Xu, 2014; Wolf et al., 2024). Numerous *in vivo* and *in vitro* studies suggest that neurotoxins, such as 1-methyl-4-phenyl-1,2,3,6-tetrahydropyridine (MPTP) and 6-OHDA, promote apoptosis in dopaminergic neurons (Dionísio et al., 2021). In our 6-OHDA-intoxicated rat model, we recorded an imbalance in the pro-BDNF/BDNF ratio. This imbalance was characterized by increased pro-BDNF expression without significant modulation of mature BDNF. Degeneration of the dopaminergic neurons in the substantia nigra is more often associated with decreased BDNF levels. However, like Chen et al., we noted an absence of BDNF modulation in the striatum after exposure to 6-OHDA despite induction of the BDNF/CREB pathway with their treatment (Chen et al., 2020). Ahmadian et al. (2018) even described significantly higher striatal BDNF levels in rats of the 6-OHDA-lesioned group than in the sham group. Our observations are also consistent with findings in the hippocampus of another model of PD in rats (Savall et al., 2023). In this study, they describe stimulation of the pro-BDNF/p75 ^NTR^ pathway without modulation of BDNF levels after exposure to MPTP. The restoration of the pro-BDNF/BDNF balance in the 6-OHDA/DCE-treated group suggests a neuroprotective impact of the treatment against apoptosis via modulation of pro-BDNF.

In conclusion, IN DCE treatment has a positive effect on motor discoordination in 6-OHDA-intoxicated rats, especially in terms of the bradykinesia, balance, and postural instability observed in this model. IN DCE treatment also reduced dopaminergic loss associated with 6-OHDA intoxication and partial restoration of neurofilaments, which are axonal markers in the striatum. The mechanism of survival or protection of dopaminergic neurons may be related to the mitigation of pro-apoptotic pro-BDNF levels. The possibility of measuring Nf-L in plasma, combined with the fact that this biomarker reflects central alterations and the impact of treatment, makes it a potential biomarker for therapeutic efficacy. Combining the ability of IN DCE to effectively supply neurons with Cr (Disdier et al., 2025) with these positive results in the 6-OHDA-lesioned rat model, IN DCE treatment might help mitigate the symptoms of patients with mitochondrial diseases.

## Data Availability

The authors confirm that the data supporting the findings of this study are available from the corresponding author on reasonable request.

## Supplementary material

SUPPLEMENTARY FIGURE 1 DCE labeled with stable isotopes (DCE-δ3).

SUPPLEMENTARY FIGURE 2 Correlation of dopamine concentration in striatum and sensorimotor performances in 6-OHDA-lesioned rats after 5 weeks of IN treatment with DCE-δ3: Ratio of dopamine concentration in the striatum of the right hemisphere (R) over the left hemisphere (L) correlated with amphetamine turning test performance (rotations per min), and with beam walking test performance (crossing time, number of segments crossed and walking score). Correlation computed by the Spearman nonparametric correlation. Red dots represent rats injected with 6-OHDA and treated with vehicle. Blue dots represent rats injected with 6-OHDA and treated with DCE-δ3. The correlation is not linear since the effect of neuronal cell loss appears mainly when 70% of the neurons are affected.
