# Supplementary material for: Intranasal delivery of dodecyl creatine ester alleviates motor deficits and increases dopamine levels in a 6-OHDA rat model of parkinsonism
Source: Front Aging Neurosci. 2025 Jul 11;17:1597263. doi: 10.3389/fnagi.2025.1597263 (PMC12289593; doi:10.3389/fnagi.2025.1597263)
Supplement: Supplementary file 1 [file Presentation_1.pptx]

## Slide 1
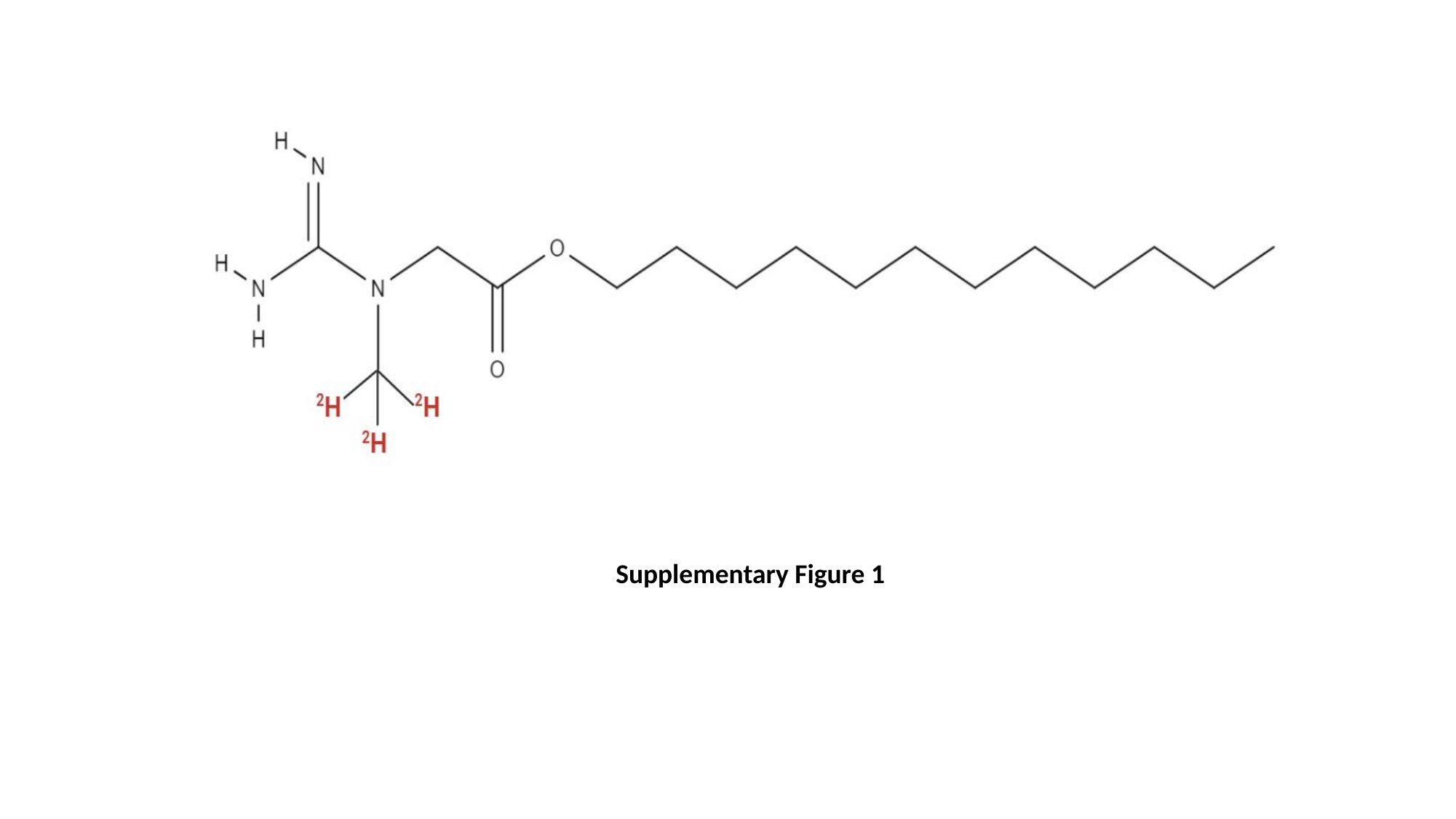

Supplementary Figure 1

## Slide 2
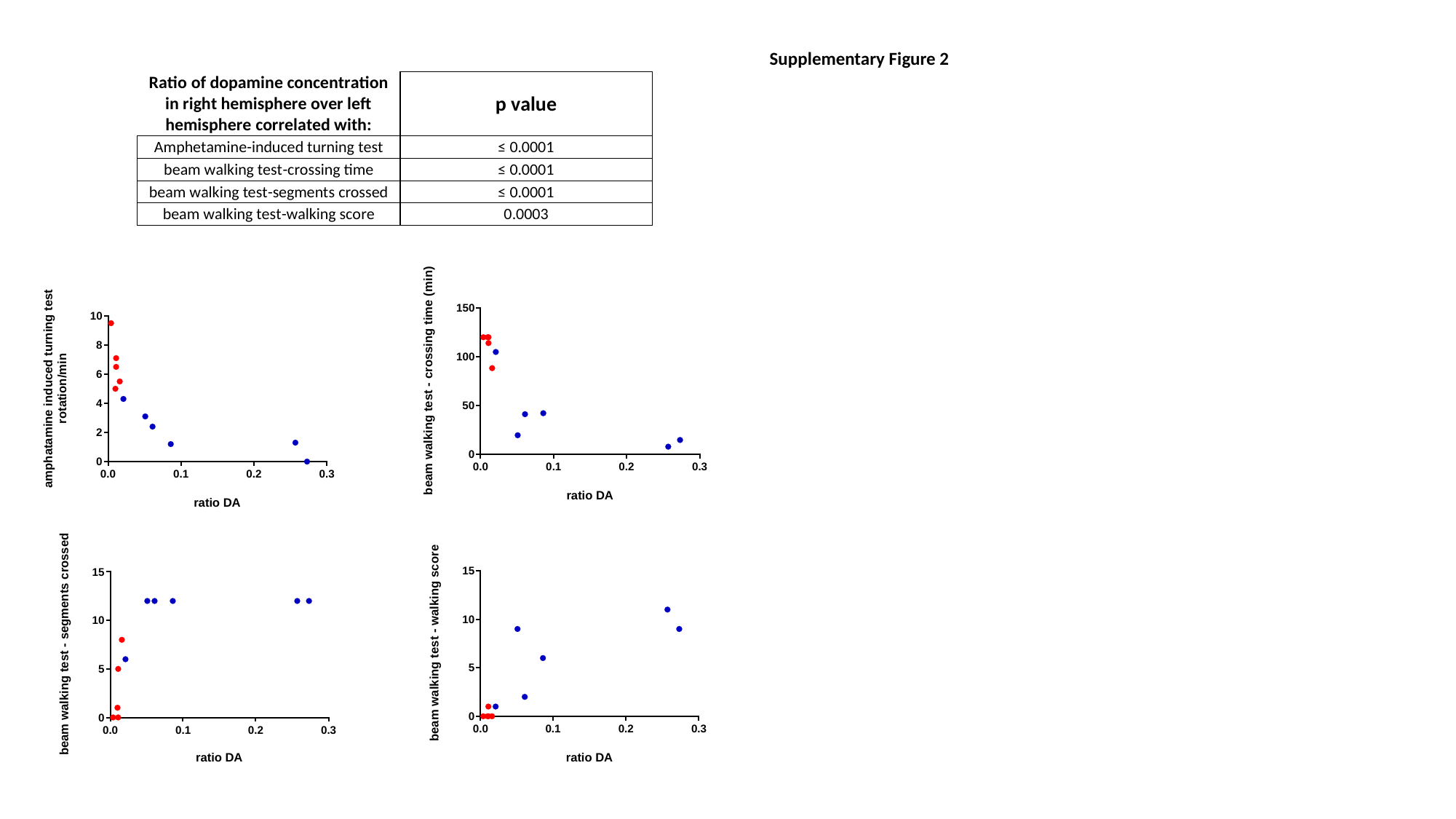

Supplementary Figure 2

## Slide 3
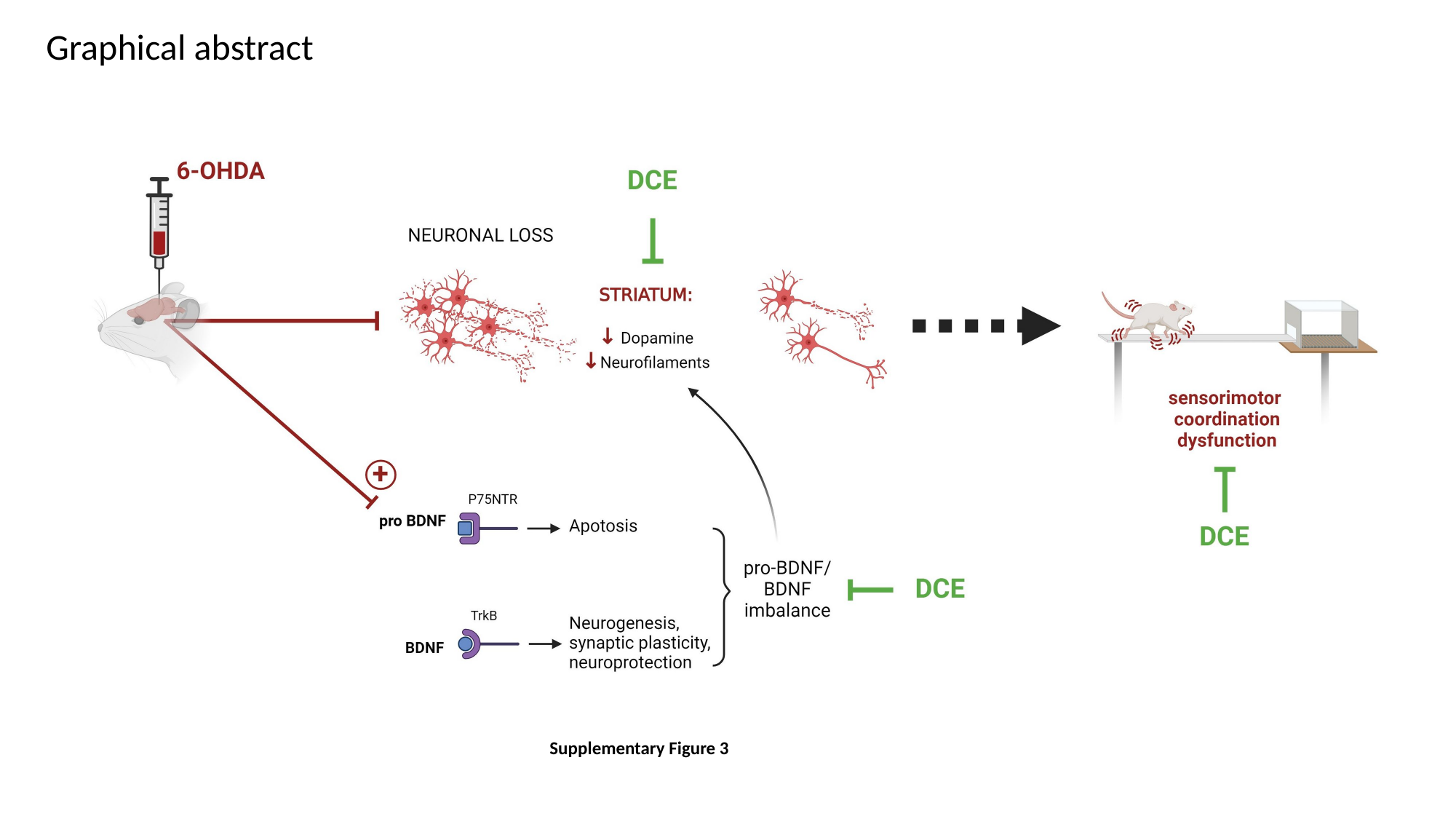

Graphical abstract
Supplementary Figure 3
